# Associations between child and adolescent marriage and reproductive outcomes in Brazil, Ecuador, the United States and Canada

**DOI:** 10.1186/s12889-022-13766-w

**Published:** 2022-07-23

**Authors:** Marcelo Luis Urquia, Rosangela Batista, Carlos Grandi, Viviane Cunha Cardoso, Fadya Orozco, Andrée-Anne Fafard St. Germain

**Affiliations:** 1 Department of Community Health Sciences, College of Medicine, Rady Faculty of Health Sciences, Manitoba Centre for Health Policy, University of Manitoba, Winnipeg, Canada; 2 Dalla Lana School of Public Health, University of Toronto, Toronto, Canada; 3 Department of Public Health, Federal University of Maranhão, São Luis, do Maranhão Brazil; 4 Ribeirão Preto Medical School, University of São Paulo, Ribeirão Preto, Brazil; 5 Universidad San Francisco de Quito USFQ, Quito, Ecuador

**Keywords:** Child marriage, Adolescent pregnancy, Preterm birth, Low birthweight, Fertility, Marital status, Brazil, Canada, Ecuador, United States

## Abstract

**Background:**

Although marriage is associated with favourable reproductive outcomes among adult women, it is not known whether the marriage advantage applies to girls (< 18 years). The contribution of girl child marriage (< 18 years) to perinatal health is understudied in the Americas.

**Methods:**

National singleton birth registrations were used to estimate the prevalence of girl child marriage among mothers in Brazil (2011–2018, *N* = 23,117,661), Ecuador (2014–2018, *N* = 1,519,168), the USA (2014–2018, *N* = 18,618,283) and Canada (2008–2018, *N* = 3,907,610). The joint associations between marital status and maternal age groups (< 18, 18–19 and 20–24 years) with preterm birth (< 37 weeks), small-for-gestational age (SGA < 10 percentile) and repeat birth were assessed with logistic regression.

**Results:**

The proportion of births to < 18-year-old mothers was 9.9% in Ecuador, 8.9% in Brazil, 1.5% in the United States and 0.9% in Canada, and marriage prevalence among < 18-year-old mothers was 3.0%, 4.8%, 3.7% and 1.7%, respectively. In fully-adjusted models, marriage was associated with lower odds of preterm birth and SGA among 20–24-year-old mothers in the four countries. Compared to unmarried 20–24-year-old women, married and unmarried < 18-year-old girls had higher odds of preterm birth in the four countries, and slightly higher odds of SGA in Brazil and Ecuador but not in the USA and Canada. In comparisons within age groups, the odds of repeat birth among < 18-year-old married mothers exceeded that of their unmarried counterparts in Ecuador [AOR: 1.99, 95%CI: 1.82, 2.18], the USA [AOR: 2.96, 95%CI: 2.79, 3.14], and Canada [AOR: 2.17, 95%CI: 1.67, 2.82], although minimally in Brazil [AOR: 1.09, 95%CI: 1.07, 1.11].

**Conclusions:**

The prevalence of births to < 18-year-old mothers varies considerably in the Americas. Girl child marriage was differentially associated with perinatal health indicators across countries, suggesting context-specific mechanisms.

## Background

Marriage is a social relationship that is associated with beneficial maternal and child health outcomes in high income countries [1–3]. The marriage advantage may stem from a beneficial influence of the marriage itself, from a selection of healthier individuals into marriage, or a combination of both [4]. Irrespective of the mechanism, most studies have confirmed this protective association in the general population mainly composed of adult women, but it is unclear whether the protective effects of adult marriage also apply to younger women, particularly among minors who have not yet achieved full citizen rights granted to adults.

Child marriage (CM), defined as a marriage or union of an individual below 18 years of age, is considered by various international agencies a violation of human rights that may negatively affect the lives, health, and future development of girls [5, 6]. Consequences of child marriage include child and teenage maternity, challenges in advancing educational and career goals, less participation in the labor market as adults, greater risk of suffering gender violence, and lack of autonomy [6, 7]. In 2015, 193 United Nations country-members agreed to end child, early and forced marriage as a means to achieve the Sustainable Development Goal of gender equality by 2030 [7].

This agenda is supported by a substantial body of literature originating from low- and middle-income countries, mainly Asia and Africa, where most early pregnancies take place within arranged marriages [6, 7]. Studies have reported negative associations between marriage before age 18 and health and social outcomes, such as lower educational attainment, limited autonomy, intimate partner violence, unintended pregnancies, higher lifetime fertility, and adverse reproductive outcomes, compared to marriage at an older age [8–14]. However, these associations may not be readily generalizable to high- and middle-income countries of the Americas, where most girl and adolescent pregnancies occur out of wedlock, non-marital births are increasingly accepted, and most marriages are believed to be consensual [15, 16]. The existence of a small proportion of child marriages in high income countries, such as the USA and Canada [17–19], raises the possibility that girls who marry early may differ from those who do not with respect to social and health characteristics. However, there remains a knowledge gap with regards to the association between child marriage and perinatal health in the Americas. Despite the abundant literature on the perinatal health of girls and adolescents, most studies have compared teen pregnancies, categorised as a single group, to those of older women. Fewer studies have distinguished subgroups within teenagers [20, 21] and the interplay between early pregnancy and marital status is not well understood.

Both the concepts of “child” and “marriage” are socially constructed entities that in practice show variation across time and space [22]. For this reason, the examination of the interplay between young maternal age and marriage and its association with reproductive outcomes may benefit from a comparative perspective, particularly in countries of the Americas where these issues remain understudied [17]. Using nationwide population-based birth registrations, including 1.57 million births to < 18-year-old mothers, we aimed to 1) quantify births to married minors in two North American and two South American countries and 2) assess the associations between maternal age and marital status with perinatal outcomes among adolescents, with emphasis on child marriage.

## Methods

### Design

This is a population-based cross-sectional comparative multi-country study. We used nationwide anonymised birth registrations available for the four countries at the time of the data analysis.

### Study populations and data sources

The study populations were composed of the most recent live births registrations in Brazil (2011–2018), Ecuador (2014–2018), United States (2014–2018) and Canada (2008–2018). The study periods are expressed in calendar years and were determined based on the availability of information on marital status, consistency in data collection over time, and subgroup size considerations. Brazilian data was obtained from the Brazilian Information System on Live Births (SINASC) through the Department of Informatics of the Unified Health System (DATASUS) [23]. Ecuadorian data was obtained from the National Institute of Statistics and Censuses (INEC) [24]. United States data was obtained from the Natality Public Use Files provided by the National Center for Health Statistics (NCHS) [25]. The Canadian Vital Statistics Live Birth Database was accessed through the Canadian Research Data Centre Network [26].

### Inclusion criteria

**Fig. 1:**
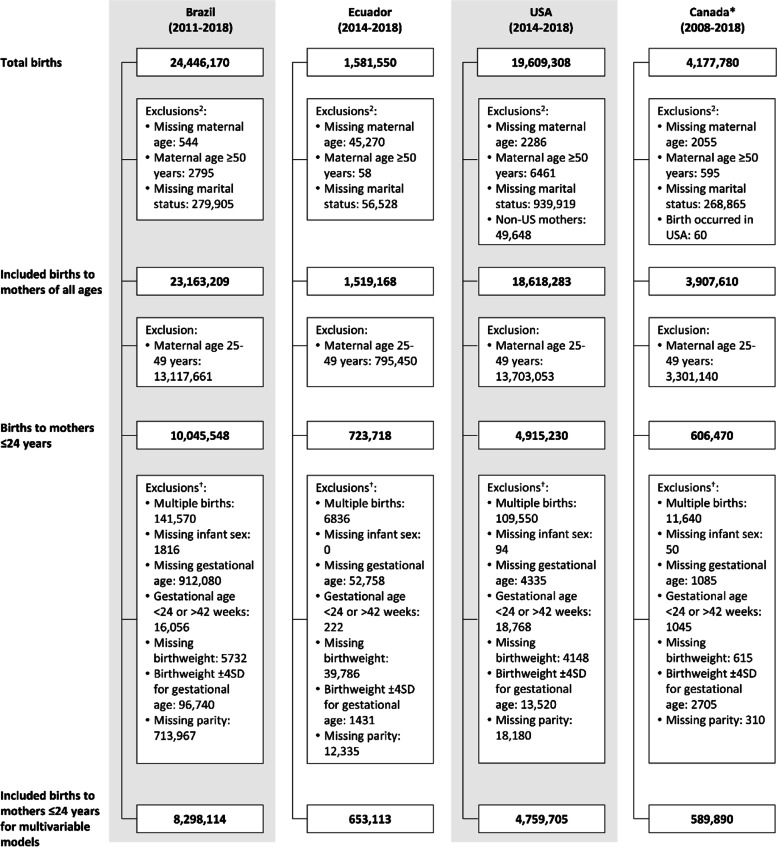
Sample selection process in Brazil, Ecuador, USA, and Canada. * To meet Statistics Canada’s confidentiality requirement, all frequencies were rounded to the nearest multiple of five using a controlled random rounding technique. † Exclusions not mutually exclusive

For our first objective of determining the distribution of births according to maternal age and marital status, we included births to mothers ≤ 49 years and excluded births with missing information on these two variables (Fig. 1).

For our second objective of examining the associations between maternal age and marital status with reproductive outcomes, we restricted the analytic sample to births of adolescent mothers ≤ 24 years, which allows to contextualise births to < 18-year-old mothers within the full range of adolescence [27, 28]. We also excluded multiple births and birth records with missing, out of range or implausible information on infant sex, gestational age, birth weight, and number of previous births. Implausible combinations of sex- and gestational age-specific birthweight were removed after detecting birthweights that were beyond four standard deviations from the sex- and gestational age-specific birthweight median based on the Intergrowth 21 international newborn standards [29]. A detailed breakdown of the exclusions is provided in Fig. 1.

### Variable definitions

#### Independent variables

In the four countries, information on marital status was self-reported by the mother and was categorised into legally married and unmarried. Divorced, widowed, and separated mothers were classified as unmarried. Common-law unions, only collected in Brazil and Ecuador, were reclassified as unmarried.

Maternal age represents the age in complete years at the time of the birth, which may differ from that of conception, and was categorised into < 18, 18–19, and 20–24 years.

#### Dependent variables

Preterm birth was defined as a birth before 37 completed weeks of gestation.

Small for gestational age (SGA) was defined as a birthweight < 10th percentile for gestational age using the sex-specific INTERGROWTH-21 birthweight charts for infants born between 24–42 completed weeks of gestation [29].

Repeat birth denotes that the current birth was preceded by one or more pregnancies resulting in a live birth.

### Data analysis

The distribution of births according to maternal age and marital status within countries was determined by cross tabulations. Logistic regression was used to model the joint associations of maternal age groups and marital status with each of the reproductive outcomes by adding a multiplicative interaction term between maternal age and marital status (3 × 2 groups). Based on the interaction model, adjusted Odds Ratios with 95% confidence intervals were estimated for the joint associations where births to unmarried mothers aged 20–24 years were the reference group. In the models of repeat birth, married women were compared to unmarried women within age group strata, because of the strong collinearity between maternal age and previous births. For preterm birth and SGA, we also compared births of married versus unmarried women within age groups but only reported the p-values in the figures while the adjusted odds ratios are provided in the text of the results section.

#### Covariates

The main model including the interaction term was run with two sets of control variables. For comparability, minimally adjusted models (Model 1) included common variables available in the four countries, infant sex, previous birth, and year of birth, where applicable. In a second model, we further adjusted for all meaningful variables to each perinatal outcome available in each country (Model 2): paternal age, maternal race, prenatal care initiated in 1st trimester, state, and age-appropriate low education in Brazil; maternal ethnicity, foreign-born mother, adequacy of the number of prenatal care visit for gestational age [30], maternal literacy, maternal region of residence, and rurality in Ecuador; paternal age, maternal race/ethnicity, foreign-born mother, any maternal smoking during pregnancy, Graduated Prenatal Care Utilization Index (GINDEX) [31], received WIC (Special Supplemental Nutrition Program for Women, Infants, and Children) during pregnancy, and delivery primarily paid by Medicaid in the USA; and paternal age, foreign-born mother, foreign-born father, province/territory of birth, reside in rural or urban area, and area-level income quintiles in Canada.

### Ethics

Brazilian, Ecuadorian and United States datasets are publicly available and therefore their use does not require review by Research Ethics Boards in their respective countries. Use of Canadian data was approved by the Canadian Research Data Centre’s Network from the Social Sciences and Humanities Research Council and by the Health Research Ethics Board of the University of Manitoba (HS24149 (H2020:356)). All methods were carried out in accordance with Statistics Canada’s vetting rules and the Helsinki Declaration.

## Results

### Distribution of births according to maternal age group and marital status

**Fig. 2:**
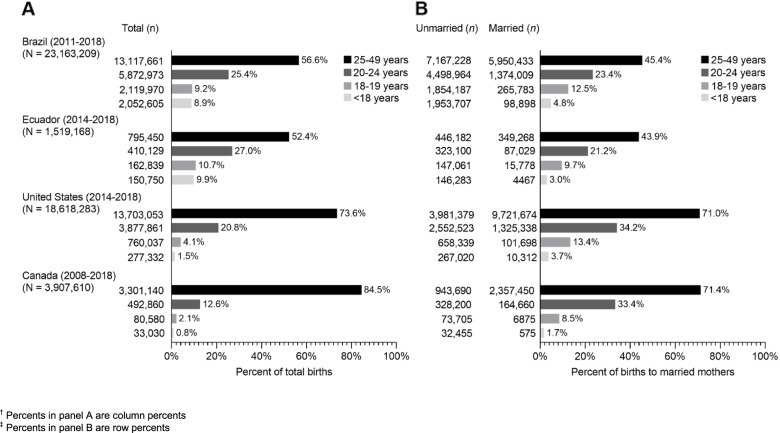
Distribution of births according to age group^†^ and married status within age groups^‡^ in Brazil, Ecuador, USA and Canada. ^†^ Percents in panel A are column percents. ^‡^ Percents in panel B are row percents

Overall, the proportion of total births increased with increasing maternal age group but varied significantly between countries. The percentage of births to mothers aged < 18 years was 9.9% in Ecuador, 8.9% in Brazil, 1.5% in the United States and 0.9% in Canada (Fig. 2, panel A).

Within age groups, the proportion of married mothers also varied between countries. More than 70% of 20–24-year-old mothers were married in the USA and Canada whereas around 45% were married in Brazil and Ecuador. Among mothers aged < 18 years, the percentage of births to legally married mothers was 4.8% in Brazil, 3.0% in Ecuador, 3.7% in the USA and 1.7% in Canada (Fig. 2, Panel B). The rate of births to married girls among all births was 42.7 per 10,000 in Brazil, 29.4 per 10,000 in Ecuador, 5.5 per 10,000 in the USA and 1.5 per 10,000 in Canada.

### Associations with reproductive outcomes

The interaction between marital status and maternal age groups was statistically significant for the three outcomes in the four countries in both models (*p*-value < 0.001), indicating that the interplay of these two variables unequivocally shapes perinatal outcomes among child and adolescent mothers.

**Fig. 3:**
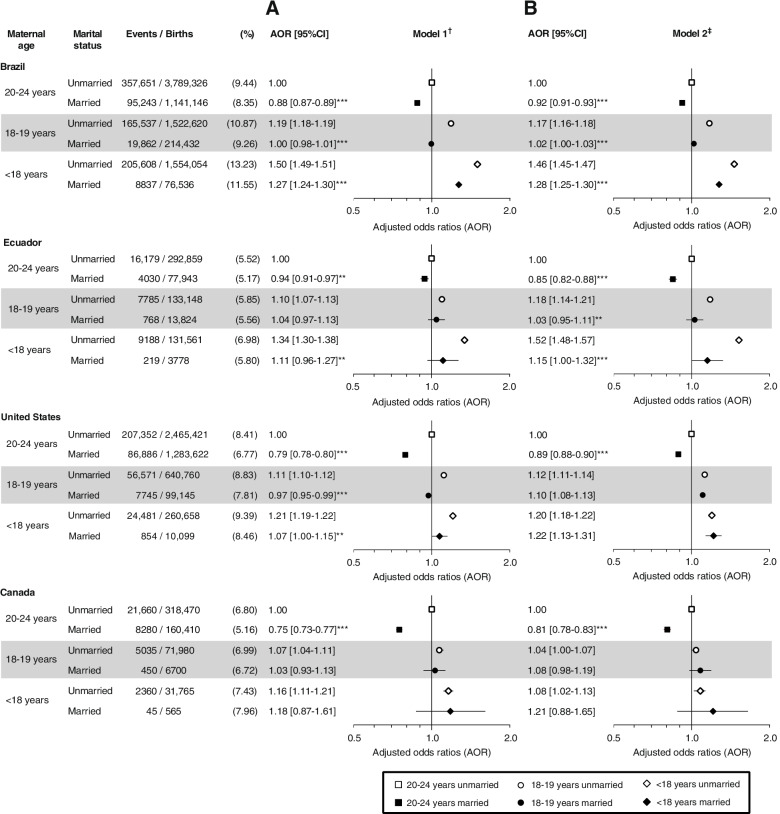
Minimally (**A**) and country-specific fully (**B**) adjusted odds ratios of preterm birth by maternal age group and marital status in Brazil, Ecuador, USA, and Canada. ^†^ Adjusted for infant sex, previous birth, and year of birth. ^‡^ Brazil: Adjusted for infant sex, previous birth, year of birth, paternal age, maternal race, prenatal care initiated in 1^st^ trimester, state, and age-appropriate low education. Ecuador: Adjusted for infant sex, previous birth, year of birth, maternal ethnicity, foreign-born mother, adequacy of the number of prenatal care visits for gestational age (WHO), maternal literacy, and maternal region of residence and rurality. USA: Adjusted for infant sex, previous birth, year of birth, paternal age, maternal race/ethnicity, foreign-born mother, any maternal smoking during pregnancy, prenatal care adequacy (GINDEX), received WIC during pregnancy, and delivery primarily paid by Medicaid. Canada: Adjusted for infant sex, previous birth, year of birth, paternal age, foreign-born mother, foreign-born father, province/territory of birth, reside in rural or urban area, and area-level income quintiles. ***p < 0.0001, **p < 0.01, *p < 0.05 for difference in odds ratios between married and unmarried mothers within age group

In the four countries, there was a gradient of increasing preterm birth rates with decreasing maternal age, for both married and unmarried mothers, being steeper in Brazil and Ecuador, particularly among unmarried mothers. Compared to unmarried mothers aged 20–24 years, both married and unmarried < 18-year-old mother had higher odds of preterm birth, although the associations were of borderline statistical significance for married girls in Ecuador, USA and Canada (Fig. 3, panel A). The odds ratio comparing married < 18-year-old with unmarried 20–24-year-old mothers increased after adding country-specific covariates in the fully adjusted models (Fig. 3, panel B), becoming statistically significant in the USA (AOR: 1.22; 95% CI: 1.13, 1.31). Compared to unmarried mothers aged 20–24 years, unmarried mothers aged 18–19 years had higher odds of preterm birth in the four countries, whereas married mothers aged 18–19 years had higher odds only in the USA, in the fully adjusted model (Fig. 3, panel B).

In comparisons within age groups, married women had consistently lower odds of preterm birth than unmarried women in the 20–24-year-old group in the four countries in the two models (*p*-values < 0.01) (Fig. 3). However, among < 18-year-old mothers, being married was associated with lower odds of preterm birth in Brazil (AOR model 1: 0.85, 95%CI: 0.83, 0.87; *p*-value < 0.0001) and in Ecuador (AOR model 1: 0.83, 95%CI: 0.72, 0.95;; *p*-value < 0.01), but not in the USA or Canada.

**Fig. 4:**
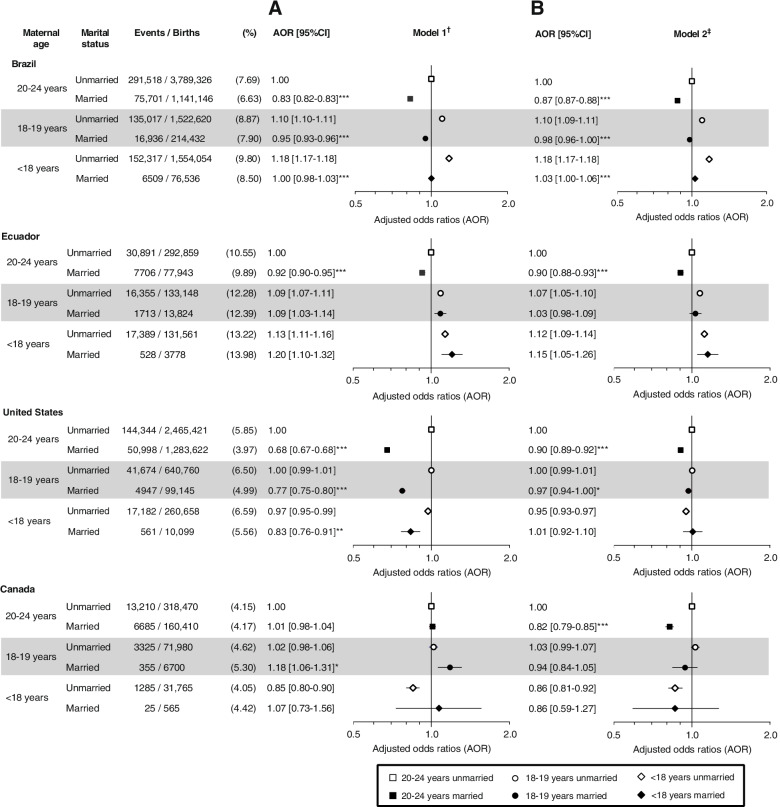
Minimally (**A**) and country-specific fully (**B**) adjusted odds ratios of small for gestational age by maternal age group and marital status in Brazil, Ecuador, USA, and Canada. ^†^ Adjusted for previous birth and year of birth. ^‡^ Brazil: Adjusted for previous birth, year of birth, paternal age, maternal race, prenatal care initiated in 1^st^ trimester, state, and age-appropriate low education. Ecuador: Adjusted for previous birth, year of birth, maternal ethnicity, foreign-born mother, adequacy of the number of prenatal care visits for gestational age (WHO), maternal literacy, and maternal region of residence and rurality. USA: Adjusted for previous birth, year of birth, paternal age, maternal race/ethnicity, foreign-born mother, any maternal smoking during pregnancy, prenatal care adequacy (GINDEX), received WIC during pregnancy, and delivery primarily paid by Medicaid. Canada: Adjusted for previous birth, year of birth, paternal age, foreign-born mother, foreign-born father, province/territory of birth, reside in rural or urban area, and area-level income quintiles. ***p < 0.0001, **p < 0.01, *p < 0.05 for difference in odds ratios between married and unmarried mothers within age group

Regarding SGA, compared with unmarried 20–24-year-old women, married < 18-year-old women only had higher odds in Ecuador but not in the other countries (Fig. 4). In Brazil and Ecuador, unmarried < 18- and 18–19-year-old mothers had higher odds of SGA than their unmarried 20–24-year-old counterparts in the two models (Fig. 4). Conversely, in the USA and Canada, unmarried < 18-year-old mothers had slightly lower odds of SGA than unmarried 20–24-year-old women in the two models, but not married mothers.

Comparisons between married and unmarried women within age groups were only consistently observed in the 20–24-year-old group. In all countries, married mothers aged 20–24 years had consistently lower odds of SGA than their unmarried counterparts, with the only exception of Canada in the minimally adjusted model (Fig. 4, Panel A). However, this association became statistically significant in the fully adjusted model (Fig. 4, Panel B). Among 18–19-year-old women, marriage was only associated with slightly lower odds in Brazil (*p*-value < 0.0001) and the USA (*p*-value < 0.05) in the fully adjusted model. Among < 18-year-old mothers, being married was only associated with lower odds of SGA in Brazil (AOR model 1: 0.85; 95%CI: 0.83, 0.87; *p*-value < 0.0001).

**Fig. 5:**
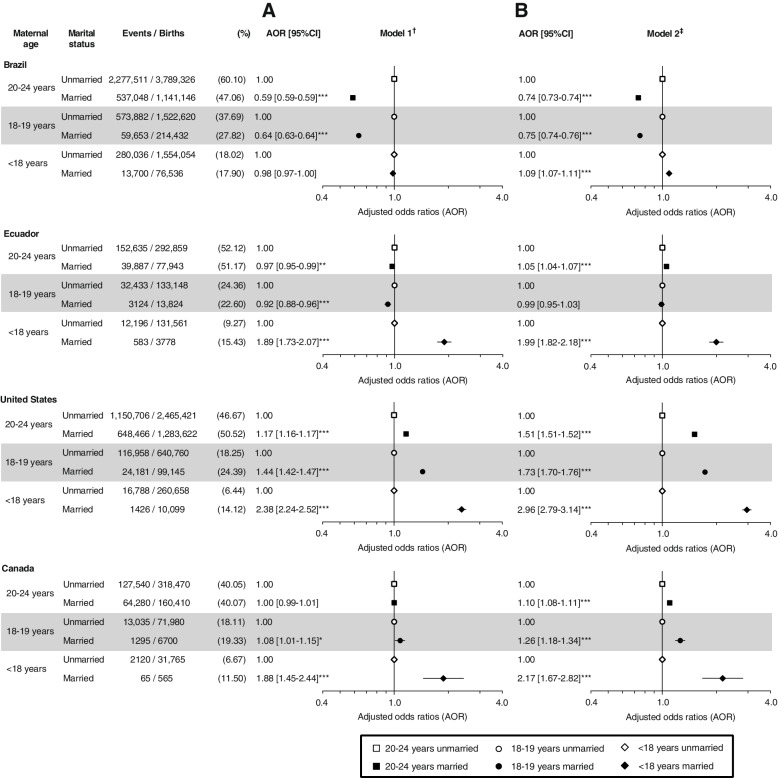
Minimally (A) and country-specific fully (B) adjusted odds ratios of repeat birth for married mothers versus unmarried mothers within maternal age group in Brazil, Ecuador, USA, and Canada. ^†^Adjusted for year of birth. ^‡^Brazil: Adjusted for year of birth, paternal age, maternal race, state, and age-appropriate low education. Ecuador: Adjusted for year of birth, maternal ethnicity, foreign-born mother, maternal literacy, and maternal region of residence and rurality. USA: Adjusted for year of birth, paternal age, maternal race/ethnicity, foreign-born mother, received WIC during pregnancy, and delivery primarily paid by Medicaid. Canada: Adjusted for year of birth, paternal age, foreign-born mother, foreign-born father, province/territory of birth, reside in rural or urban area, and area-level income quintiles. ***p < 0.0001, **p < 0.01, *p < 0.05 for difference in odds ratios between married and unmarried mothers within age group

Unlike preterm birth and SGA, comparisons of repeat birth by marriage status were restricted within age group strata (Fig. 5) because the likelihood of previous births is strongly colinear with age. Unlike Brazil and Ecuador, married women in the USA and Canada had higher odds of repeat birth than unmarried women in all age groups. In all countries, the highest odds of repeat birth were observed among married mothers aged < 18 years relative to their unmarried counterparts in the two models. The association was two- to three-fold in all countries, except in Brazil, where a weak association was only present in the fully adjusted model (Fig. 5, Panel B). In the USA and Canada marriage was associated with higher odds of repeat birth among 18–19- and 20–24-year-old mothers. However, the pattern was reversed Brazil, with lower odds among married 18–19- and 20–24-year-old women, and no significant difference in Ecuador.

## Discussion

### Main findings

This cross-country population-based study indicates that the frequency of child marriage varies substantially in the Americas, from 1.5 per 10,000 births in Canada to 42.7 per 10,000 births in Brazil. Our main finding is that among girl and adolescent mothers, age and marital status interact to shape reproductive outcomes. Furthermore, we found that the interplay between age and marital status is context-dependent, as evidenced by differential patterns between countries. The well-documented perinatal health advantage associated with adult marriage was confirmed among births to mothers aged 20–24 years but was not consistently observed among births to 18–19-year-old and < 18-year-old mothers. The protective association of marriage with preterm birth among < 18-year-old mothers in Brazil and Ecuador was offset by increased odds associated with decreasing maternal age. Child marriage was strongly associated with repeat birth in all countries, except in Brazil, where marriage was also associated with lower odds of repeat birth among 18–19- and 20–24- year-old mothers.

### Interpretation

Our study confirms the advantage of marriage among 20–24-year-old mothers, as documented for preterm birth, SGA and other perinatal outcomes [1, 3, 4]. This beneficial association has been well documented among all women but has not been examined in detail among younger mothers in high-income countries, particularly among those below age 18. We found that the protective effect of marriage observed among adult women was weakened among those aged 18–19 and < 18 years, if not absent, and when present, such as in the case of preterm birth, it was offset by the higher odds associated with an early age. This modification of the association of marriage with decreasing age suggests that the mechanisms by which marriage influences health may not be the same for adult women and girls. While the increasing gradient in preterm birth associated with decreasing age may reflect biological and social immaturity for childbearing, marital status differences within age groups that reflect the influence of social contexts may not be strong enough to counterbalance the age gradient. The marriage advantage, generally observed in the general adult population, is thought to result from providing a context conducive to healthier behaviors (e.g., lower tobacco and alcohol consumption) that translate in better health, from a selection of healthier individuals into marriage (e.g., higher income, wealth, education, race-ethnicity) or a combination of both [4]. Underage marriage may not be as protective as adult marriage due to deeper gender inequities, manifested as power imbalance, lack of autonomy, and financial dependence [6, 7]. In addition, selection mechanisms into marriage may be different between age groups and not necessarily confer protection to minors, such as marriage pressured by family members driven by religious beliefs, urgency to legitimise a pregnancy, or marriage to escape poverty or an abusive family environment [16]. Since different pathways may be operating in various degrees in the four countries and beyond, further longitudinal research may be valuable.

Higher odds of repeat birth among married women in all age groups in the USA and Canada may simply reflect intended pregnancies towards the goal of family formation. However, married < 18-year-old women may have limited ability to negotiate contraceptive use and sexual intercourse frequency resulting in unintended high early fertility [10]. Interestingly, the strongest association between marriage and repeat birth was among < 18-year-old women in all countries, except in Brazil. Giving birth to multiple children at an early age may undermine girls’ ability of self-development, which in turn may affect their capability to provide optimal care to their children [6]. Repeated pregnancy among teenagers may also be associated with short interpregnancy intervals and a higher risk of preterm delivery and stillbirth in subsequent pregnancies [32]. The exception of lower repeat birth rates among 18–19- and 20–24-year-old married mothers in Brazil may be due to delayed childbearing within marriage or to planning of small families associated with higher socioeconomic status. Overall fertility trends have reached below replacement levels in Brazil, particularly among the well-off, but remain higher among women residing in poor regions, of low education, and of non-white skin color [33].

### Limitations

There are a number of limitations. First, self-reported marital status within pre-established categories [15] may have resulted in some degree of misclassification. Informal unions are not collected in the USA and Canada, and therefore we restricted analyses to categories comparable across countries, resulting in the classification of informal unions as unmarried in Brazil and Ecuador. This limitation constrained us to focus on legal marital status (legally married versus unmarried). Varying proportions of informal unions in the four countries may have biased comparisons towards the null, since adverse perinatal outcomes of common-law women are intermediate between those of legally married and single never married women [2, 3]. Second, cross-sectional data lacking the date of marriage cannot be used to discriminate whether marriage preceded conception or occurred during pregnancy. Third, since birth registrations occur at or after the birth of the child, many births to 18-year-old mothers may have been conceived at 17 years of age and contributing to an underestimation of pregnancies of minor mothers. Fourth, birth registrations do not contain a maternal identifier to help relate different births of the same mother over time. Therefore, it was not possible to determine if women who gave birth to a second or third child after 18 years of age also gave birth before turning 18. Finally, an unknown degree of residual confounding may be present due to the availability of variables and measurement error. Despite some common patterns across countries (marriage advantage among 20–24-year-old mothers, age gradient in preterm birth and SGA), there were country-specific patterns that may not be generalizable to other countries of the Americas, Europe and the rest of the world, which raises the need of further empirical studies that clarify how age and marital status interact in among adolescents in different settings.

## Conclusions

Despite the abovementioned limitations, this study provides a comparative view of the differential reproductive outcomes of married and unmarried girls and adolescent women in four American countries with different socioeconomic contexts and rates of girl child marriage. Among adolescents aged < 25 years, an interplay between maternal age and marital status shaping reproductive outcomes was observed in all countries but the patterns were different. This observation stresses the context-dependent nature of the joint influence of maternal age and marital arrangements on reproductive outcomes.

## Data Availability

Brazilian, Ecuadorian and United States data are publicly available. Brazilian data was obtained from the Brazilian Information System on Live Births (SINASC) through the Department of Informatics of the Unified Health System (DATASUS) https://datasus.saude.gov.br/. Ecuadorian data was obtained from the National Institute of Statistics and Censuses (INEC) https://www.ecuadorencifras.gob.ec/nacimientos-bases-de-datos/. United States data was obtained from the Natality Public Use Files provided by the National Center for Health Statistics (NCHS) https://www.cdc.gov/nchs/data_access/vitalstatsonline.htm. Canadian data are available on reasonable request and available from Statistics Canada for researchers who meet the criteria for access to confidential data (contact Statistics Canada Regional Data Centres at https://www.statcan.gc.ca/eng/microdata/data-centres/access). The Canadian Vital Statistics Live Birth Database was accessed through the Canadian Research Data Centre Network https://www23.statcan.gc.ca/imdb/p2SV.pl?Function=getSurvey&SDDS=3231.

## Abbreviations

AOR: Adjusted odds ratio
CM: Child marriage
DATASUS: Department of Informatics of the Unified Health System (Brazil)
GINDEX: Graduated Prenatal Care Utilization Index
INEC: National Institute of Statistics and Censuses (Ecuador)
NCHS: National Center for Health Statistics
SGA: Small-for-gestational age
SINASC: Brazilian Information System on Live Births
USA: United States of America
WIC: Special Supplemental Nutrition Program for Women, Infants, and Children
